# Effects of Crushing, Vacuum Nano-Collision, and Steam Explosion on the Flavor and Physical Properties of Solid Spices

**DOI:** 10.3390/foods14234010

**Published:** 2025-11-22

**Authors:** Kunyang Chen, Dezi Zhang, Yanxia Liu, Yaodi Zhu, Miaoyun Li, Lijun Zhao, Fukang Dong, Gaiming Zhao, Niancheng Hong, Shijie Liu, Shiru Du

**Affiliations:** 1 College of Food Science and Technology, Henan Agricultural University, Zhengzhou 450002, China; 15037078410@163.com (K.C.);; 2 Sauce Braised and Prefabricated Products Modern Production School Enterprise Research and Development Center, Henan Agricultural University, Zhengzhou 450002, China; 3 Henan Jiuyuquan Food Co., Ltd., Postdoctoral Innovation Base, Yuanyang 453500, China

**Keywords:** GC-MS, UPLC-MS, culinary spices, processing methods, key flavor compounds, major bioactive components

## Abstract

Spices play a crucial role in shaping the characteristic flavor of marinated meat products. This study systematically compared the effects of physical crushing, vacuum nano-collision, and steam explosion on the physical and flavor characteristics of star anise and cinnamon. The vacuum nano-collision treatment effectively reduced particle size to below 15 nm, promoting faster flavor release and improving both moisture retention and solubility. Hydrocarbons, alcohols, and aldehydes were identified as the dominant volatile compounds. Among the non-volatile components, crushed cinnamon contained the highest shikimic acid concentration (1510.1 ± 25.45 μg/kg), while star anise treated with vacuum nano-collision reached the highest level of shikimic acid (893.10 ± 23.99 μg/kg). However, the main active components of these two spices did not show significant differences between the two treatment methods. Steam explosion treatment resulted in the lowest levels of both volatile and non-volatile compounds. Flavor profiling and electronic tongue analyses further revealed that the flavor characteristics of the crushed and nano-collision groups were similar, but distinctly different from those obtained with steam explosion. Overall, these results provide new insights into the development of efficient spice processing technologies and offer practical guidance for optimizing flavor quality in marinated meat products.

## 1. Introduction

Spices are extensively used in the food industry for their ability to enhance flavor and aroma. Natural spices refer to specific parts of plants—such as leaves, stems, flowers, seeds, fruits, grains, bark, roots, and bulbs—that naturally contain distinctive sensory properties. These components, or the whole plant, are commonly employed as seasonings or flavoring agents in a wide range of culinary applications [1]. Spices have been widely used since ancient times and have become an essential component of the dietary practices of the majority of populations worldwide [2,3]. Spices are indispensable auxiliary materials in meat processing, capable of enhancing meat flavor and possessing antimicrobial, preservative, and antioxidant properties [4,5]. Spices not only enhance food flavor but also—through their diverse colors, such as black, green, red, and brown—provide a true sensory experience that directly influences consumer appetite [6]. Certain traditional spices can improve the flavor of marinated meat products, among which star anise is indispensable [7,8]. Star anise is an important medicinal and edible plant in China, containing low-molecular-weight alcohols, ethers, esters, and other compounds [9]. As an indispensable ingredient in marinated meat products, star anise imparts a unique flavor to the meat. Its bioactive compounds include terpenes, alkaloids, flavonoids, phenolic compounds, and others [10]. For example, cinnamon contains compounds such as nonanal, acetic acid, and coumarin, which can enhance the aroma of meat [11]. The main components of Sichuan peppercorn include linalool, linalyl acetate, and limonene, which are key contributors to its aroma and flavor [12]. Zhong et al. investigated the contribution of star anise to the aroma of braised duck legs and found 17 key aroma compounds with odor activity values (OAV) greater than 1. These compounds include aldehydes, alcohols, ketones, furans, hydrocarbons, and ethers, which impart distinctive floral and herbal notes characteristic of braised duck legs [13]. Cinnamon is also one of the main spices used in marinated meat products. Historically, cinnamon has been highly valued across various cultures for its culinary applications [14]. In Xing et al.’s study [11], the flavor profile of cinnamon was characterized using HS-SPME-GC/MS technology, which identified seven key compounds in the cinnamon extract: nonanal, acetic acid, α-cubebene, benzaldehyde, ethyl cinnamate, trans-cinnamaldehyde, and coumarin.

Currently, common processing methods for spices include grinding them into powder or extracting their essential oils for use in marinated meat products. In Zhu et al.’s study [15], the application of spice essential oils to chicken breast revealed that cinnamon essential oil exhibited the lowest minimum inhibitory concentration against *Clostridium perfringens*. This finding contributes to controlling and rapidly assessing the food safety risk posed by *C. perfringens* during meat processing.

Currently, spices in marinated meat products are predominantly added in their solid form during the stewing process. While this method imparts distinctive flavor and color to the meat products, it results in low utilization efficiency of the spices, as a significant portion of spice components are not fully released during stewing, leading to resource waste. Additionally, the leftover spice residues after stewing, if improperly handled, can cause environmental pollution and increase disposal costs, thereby limiting industrial development to some extent. Vacuum nano-collision technology is an emerging method for spice processing. The process first grinds solid spice particles into powder, followed by cryogenic milling using a colloid mill to achieve nanoscale particle size. During milling, the temperature is carefully controlled using a circulating cooling system to maintain 5–10 °C, and the material is pre-cooled to 5 °C. Intermittent milling is applied to prevent excessive heat buildup, minimizing the loss of volatile flavor compounds while achieving uniform particle size reduction. Subsequently, a spice-to-water ratio of 1:5 is subjected to ultra-high-pressure vacuum nano-collision treatment at 170–200 MPa, producing a novel water-soluble spice with a dispersed, stable microemulsion or sub-microemulsion structure. This technology significantly improves the extraction efficiency and flavor release rate of spices while preventing residue from being left behind after use, a problem that is common with traditional spices. It offers cleanliness, environmental friendliness, energy efficiency, and a high yield rate. However, few reports exist on the use of spices processed in this way in marinated meat products, and it is unclear to what extent flavor is lost during the emulsification of solid spices.

This study investigates the effects of three different processing methods—traditional grinding, vacuum nano-collision, and steam explosion—on the physical properties and key flavor compounds of two commonly used spices in braised meat products: star anise and cinnamon. The particle size, solubility, and moisture content of the processed spices were analyzed. Gas chromatography–mass spectrometry (GC-MS) was employed to qualitatively and quantitatively analyze the volatile flavor components under each treatment. Meanwhile, liquid chromatography–mass spectrometry (LC-MS) was used to determine the changes in the content of two representative non-volatile active flavor compounds—shikimic acid in star anise and cinnamaldehyde in cinnamon. Additionally, an electronic tongue was applied to evaluate the taste differences in the braised broth prepared with the processed spices. This research aims to elucidate the influence of different processing methods on the composition and content of flavor substances in spices, thereby providing a theoretical basis for improving the flavor release efficiency and product flavor stability. The findings may also serve as a reference for the industrial production of braised meat products with low carbon emissions and low energy consumption.

## 2. Materials and Methods

### 2.1. Experimental Materials and Reagents

Star anise and cinnamon (Jiuduo Group Food Co., Ltd. Xinxiang, Henan Province, China). n-Hexane (chromatographic grade) (Shanghai Miclin Biotechnology Co., Ltd. (Shanghai, China). 2-Methyl-3-heptanone (purity 99%), shikimic acid (purity ≥ 98%), and cinnamaldehyde standard (purity ≥ 98%) were purchased from Shanghai Macklin Biochemical Technology Co., Ltd.

### 2.2. Major Instruments and Equipment

The instruments used were Ultra-Vacuum Nano-Collision Processor SVNC-P180X (Wuxi Chaona Instrument Technology Co., Ltd. (Wuxi, Jiangsu Province, China), Steam Explosion Apparatus QB-S-80B ((Henan Zhengzhou Qibao Environmental Protection Technology Co., Ltd. (Zhengzhou, Henan Province, China), Grinder, Colorimeter Model SR-60, and Centrifuge Neo 1600R (Shanghai Diyi Scientific Instrument Co., Ltd., Shanghai, China), Vortex Mixer and Electronic Balance Model JJ500 (Changshu Shuangjie Testing Instrument Co., Ltd., Changshu, China), Electronic Tongue System (Suzhou Baoman Precision Instrument Co., Ltd., Suzhou, Jiangsu Province, China), Ultrasonic Cleaner Model SYU-10-200DT (Zhengzhou Shengyuan Instrument Co., Ltd., Zhengzhou, Henan Province, China), Gas Chromatograph–Mass Spectrometer, Agilent 7010D Triple Quadrupole (Agilent Technologies, Santa Clara, CA, USA), Liquid Chromatograph (Agilent Technologies, USA), and Mass Spectrometer (Thermo Fisher Scientific, Waltham, MA, USA).

### 2.3. Materials and Methods

#### 2.3.1. Vacuum Nano-Collision Method

Solid spice particles were ground into powder and mixed with water at a ratio of 1:5. The mixture was subsequently milled using a graded colloid mill and then subjected to ultra-vacuum nano-collision at a pressure of 170–200 MPa, resulting in a uniformly dispersed and structurally stable microemulsion or sub-microemulsion system.

#### 2.3.2. Steam Explosion Treatment

The spices were pulverized to a particle size of 100 mesh and placed into the feed inlet of the steam explosion apparatus, which was then tightly sealed. After introducing compressed steam, the power supply was switched on and the pressure was gradually increased to approximately 2 MPa. The steam explosion duration was set to 30 s. Following the release of steam and dissipation of vapor, the treated material was collected. The process was repeated until the sample reached a paste-like consistency, after which the final samples were collected for further analysis.

#### 2.3.3. Natural Solid Spice Treatment Method

An appropriate amount of natural solid spices was weighed and placed into a grinder. The lid was securely fastened, and the power was switched on. The grinding process was carried out in five cycles, with the switch operated at intervals of approximately 5 s. The ground samples were then collected for further use.

#### 2.3.4. Moisture Content Analysis of Vacuum Nano-Collision Treated Spices

The moisture content was determined according to the method of Mohammadi-Moghaddam et al. [16], combined with the Chinese national standard GB 5009.3-2016, with slight modifications. The oven-drying method was employed. First, the weight of an empty aluminum dish was measured and recorded. Approximately 10 g of liquid spice sample was then weighed into the dish, with three replicates prepared and recorded. The samples were dried in an oven at 110 °C for 8 h, removed, and reweighed. Moisture content of the liquid spice samples was calculated using the standard formula, the moisture content was calculated using the following equation:

$$W=\frac{m_{1}-m_{2}}{m_{1}-m_{3}}$$

where *W* represents the moisture content of the sample; *m*_1_ is the mass of the weighing dish and sample before drying (g); *m*_2_ is the mass of the weighing dish and sample after drying (g); and *m*_3_ is the mass of the empty weighing dish (g). Results were expressed as the mean of three replicates and reported to two decimal places.

#### 2.3.5. Solubility Analysis of Spices

The water-soluble index (WSI) of the spices was determined following the procedure described by Li et al., with slight modifications [17]. Approximately 5 g of the weighed spice sample was placed into a centrifuge tube, and 100 g of distilled water was added, giving a total mass of M_0_. The mixture was subjected to ultrasonic treatment at 40 °C and 800 W for 1 h, followed by vortex mixing for 30 min. The sample was then centrifuged at 10,000× *g* rpm and 4 °C for 20 min. The supernatant was carefully decanted into a beaker, and the remaining solid residue was collected and weighed as M_1_. The calculation was performed as follows:

$$WSI=\left(1-\frac{M_{1}}{M_{0}}\right)\times 100\%$$

where *WSI* represents the solubility of the spice, M_0_ is the total mass of the spice and water, and M_1_ is the mass of the remaining solid residue.

#### 2.3.6. Determination of Spice Particle Size

According to the method of Zhao et al. [18], the prepared spice samples were measured for particle size, reported as particle number percentage (%) and average particle diameter (nm). Samples were diluted to 1.0 mg/mL with PBS (10 mmol/L, pH 7.2–7.4) and analyzed at 20 °C using a Zetasizer Pro (Malvern PANalytical Limited, Shanghai, China).

#### 2.3.7. GC-MS Analysis

##### Sample Preparation

For each of the three types of star anise and cinnamon, 4 g of the sample was weighed into a centrifuge tube. Then, 40 mL of n-hexane and 40 μL of 2-methyl-3-heptanone solution were added. The mixture was vortexed for 10 min, followed by ultrasonic treatment at 800 W for 60 min. After ultrasonication, the samples were centrifuged at 10,000× *g* rpm and 4 °C for 10 min. The extract was then filtered, and the supernatant was passed through a 0.22 μm microporous filter into a vial for GC-MS analysis.

##### GC-MS Chromatographic Conditions

GC analysis was carried out using an Agilent DB-17 capillary column. High-purity helium served as the carrier gas. The injector was maintained at 263 °C, with a column flow rate of 1.5 mL/min and a purge flow of 8 mL/min. Samples were injected in split mode (split ratio 10:1). The oven temperature program was: 50 °C initially, increased at 10 °C/min to 120 °C, then 5 °C/min to 200 °C, and finally 5 °C/min to 263 °C, held for 10 min. Injection volume was 1 μL with a split ratio of 50:1.

##### Mass Spectrometry Conditions

Electron ionization (EI) was used as the ionization source with an ionization energy of 70 eV. The ion source temperature was kept at 230 °C, while the injector temperature and auxiliary heater temperature were set at 300 °C and 280 °C, respectively. A solvent delay of 3 min was applied during the analysis.

##### Analysis of Volatile Flavor Compounds

Qualitative Analysis of Volatile Compounds: The retention times and mass spectra of the compounds were compared with those reported in relevant literature and the NIST08 mass spectral library, with all compounds achieving match scores of 85% or higher.

Quantitative Analysis of Volatile Compounds: According to the method of Sun et al. [19], 2-Methyl-3-heptanone was used as the internal standard, and the content of volatile compounds was calculated according to Equation (1).

(1) $$P=\frac{C_{0}\times S}{S_{0}}$$

where *P* represents the content of the volatile compound (mg/kg), *C*_0_ is the concentration of the internal standard (mg/kg), *S* is the peak area of the volatile flavor compound, and *S*_0_ is the peak area of the internal standard.

Evaluation of Key Volatile Flavor Compounds: The odor activity value (OAV) was used as an important indicator to evaluate the contribution of a specific aroma compound to the overall aroma profile of a food. It reflects the intensity or contribution of a particular aroma compound in a given food matrix. According to the method of Wang et al. [20], the calculation was performed according to Equation (2).

(2) $$OVA=\frac{P}{T}$$

where *P* represents the content of the volatile compound (mg/kg), and *T* is the threshold of the aroma compound in water (mg/kg), as obtained from the literature [21,22,23,24,25,26]. The thresholds were also obtained from the online database (http://www.odour.org.uk, accessed on 16 May 2025). A higher OAV indicates a greater contribution of the aroma compound to the overall aroma profile of the food, reflecting its significance in the food’s aroma system. Generally, compounds with OAV > 1 are considered major contributors to food aroma, whereas those with OAV < 1 have a negligible impact on the overall flavor [27].

#### 2.3.8. HPLC-MS Analysis

##### Preparation of Sample Solution

Approximately 1.0 g of the sample was accurately weighed into a 250 mL Erlenmeyer flask, to which 50 mL of methanol was added. The flask was weighed and subjected to ultrasonic extraction for 20 min, followed by standing for 5 h. A second ultrasonic treatment was then performed, after which the flask was reweighed, and any solvent loss was compensated for with methanol. Subsequently, a 1.0 mL aliquot of the extract was transferred into a 25 mL volumetric flask, diluted to volume with methanol, and mixed thoroughly to obtain the sample solution for analysis.

##### Preparation of Standard Solution

A standard stock solution of cinnamaldehyde was prepared by accurately weighing 20.824 mg of the reference standard into a 20 mL volumetric flask and diluting to the mark with methanol, followed by thorough mixing. Aliquots of 0.01, 0.2, 0.4, 0.6, 0.8, and 1.0 mL of the stock solution were transferred into 10 mL volumetric flasks and diluted to volume with methanol to obtain working solutions at concentrations of 1.081, 5.405, 10.81, 21.62, 54.05, and 108.1 μg·mL^−1^, respectively. Subsequently, 10 μL of each working solution was injected for analysis. A calibration curve was constructed by plotting the peak area (Y) against the corresponding concentration of cinnamaldehyde (X), and the linear regression equation was established.

A standard stock solution of shikimic acid was prepared by accurately weighing 20.824 mg of the reference standard into a 20 mL volumetric flask and diluting it to the mark with methanol, followed by thorough mixing. Aliquots of 0.01, 0.2, 0.4, 0.6, 0.8, and 1.0 mL of the stock solution were transferred into 10 mL volumetric flasks and diluted to volume with methanol to create working solutions with concentrations of 1.081, 5.405, 10.81, 21.62, 54.05, and 108.1 μg/mL, respectively. Then, 10 μL of each working solution was injected for analysis. A calibration curve was generated by plotting the peak area (Y) against the corresponding concentration of shikimic acid (X), and the linear regression equation was established.

##### Chromatographic Conditions

The chromatographic conditions were established with reference to the method described by Yang et al. [28], with slight modifications. Chromatographic separation was performed on an Agilent SB-ZORBAX C18 column (250 mm × 4.6 mm). The mobile phase consisted of solvent A and solvent B. In the positive ion mode, solvent A was 0.1% (*v*/*v*) formic acid in water, while in the negative ion mode, solvent A was 5 mmol/L ammonium acetate in water. In both modes, solvent B was acetonitrile. A binary gradient elution program was applied as follows: 0 min, 1% B; 1 min, 1% B; 8 min, 99% B; 10 min, 99% B; 10.1 min, 1% B; 12 min, 1% B. The flow rate was set at 0.5 mL/min, and the injection volume was 2 μL.

##### Mass Spectrometry Conditions

The mass spectrometric parameters were set as follows: the spray voltage was +3.8 kV in positive ion mode (POS) and −3.1 kV in negative ion mode (NEG); the capillary temperature was maintained at 320 °C. The sheath gas and auxiliary gas flow rates were set at 10 and 15 arb, respectively. The scan range was *m*/*z* 300–1500. The resolution was 70,000 for full MS scans and 17,500 for MS/MS scans. Collision energies were applied at 15, 30, and 45 eV.

### 2.4. Electronic Tongue Analysis of Braised Chicken with Spices

#### Sample Preparation of Chicken Meat

The procedure was performed with reference to Zhan et al. [29]. The marinated chicken drumsticks were homogenized, and approximately 20 g of the sample was transferred to a 250 mL conical flask. For each sample, 200 mL of ultrapure water was added, and the mixture was incubated in a water bath at 50 °C for 20 min. Subsequently, the sample was centrifuged at 10,000× *g* rpm and 4 °C for 10 min. The resulting supernatant was collected, filtered through a 0.45 μm microporous membrane, and then subjected to electronic tongue analysis. The system operates using an array of biomimetic sensors that exhibit cross-sensitive responses to specific taste substances. These biomimetic sensors are designed to simulate the human gustatory system, where each sensor mimics the response of human taste receptors to basic tastes such as sourness, saltiness, umami, sweetness, and bitterness. Unlike traditional chemical sensors that respond to a single analyte, biomimetic sensors show partial selectivity and generate characteristic potential patterns when exposed to complex food matrices. During measurement, the sensors were immersed in the sample extract, and changes in membrane potential were recorded, reflecting the overall taste characteristics of the samples. Subsequently, principal component analysis (PCA) was applied to the collected multidimensional raw data to distinguish the overall taste differences among the different sample groups.

### 2.5. Data Analysis

Data analysis was conducted using SPSS 27.0 software. Origin 2024 was employed to generate principal component analysis (PCA) and cluster heatmaps. To ensure analytical accuracy, differences and variations in flavor components among groups were examined using SIMCA14.1 software (MKS Data Analytics Solutions, Umea, Sweden). MetaboAnalyst was also utilized for threshold plotting and correlation analysis of flavor components.

## 3. Results and Discussion

### 3.1. Impact of Processing Methods on Spice Physical Properties

#### 3.1.1. Analysis of Moisture Content in Spices

In this study, the moisture content of natural solid spices (star anise and cinnamon) subjected to vacuum nano-collision and steam explosion treatments was measured to assess the impact of these processing methods on the water characteristics of the spices. As shown in Table 1, star anise (Lsa) and cinnamon (Lc) treated by vacuum nano-collision showed higher moisture content compared to those treated by steam explosion (Sea and Sec), with values exceeding 75%. Further analysis revealed that spices processed through vacuum nano-collision had a solid-to-liquid ratio of about 1:3 to 1:4, meaning that 1 kg of solid spice could produce approximately 3–4 kg of liquid spice, resulting in over 300% of the original product. These findings demonstrate that spices processed with this technique not only retain higher moisture but also achieve greater production efficiency, highlighting their resourcefulness and economic advantages in industrial applications.

#### 3.1.2. Analysis of Spice Solubility

The solubility of spices prepared with different processing methods was evaluated, and the results are shown in Table 1. Star anise (Lsa) and cinnamon (Lc) treated with vacuum nano-collision showed solubility values exceeding 90%, indicating excellent dissolution properties. In contrast, spices processed by the other two techniques had significantly lower solubility. Spices treated with vacuum nano-collision technology dissolve quickly and release their active components efficiently, enabling better interaction with food ingredients and significantly speeding up flavor release. Meanwhile, spices prepared using the other two methods dissolve more slowly. They are less evenly distributed in the food matrix, resulting in uneven flavor release and negatively impacting the overall taste and sensory quality of the final dish.

#### 3.1.3. Analysis of Spice Particle Size

The particle size of spices has a significant effect on the release of their volatile compounds. Generally, smaller particle sizes result in higher solubility, which facilitates more efficient release of volatile components. In this study, the particle sizes of spices treated by nano-collision technology and steam explosion were measured, and the results are presented in Table 1. Spices subjected to nano-collision treatment reached the nanoscale, with all particles having diameters below 15 nm. Although spices treated by steam explosion also reached the nanoscale, their particle sizes were considerably larger than those obtained by nano-collision. Previous studies have shown that reducing the particle size of *Astragalus* powder from 37 μm to 7.56 μm not only improved its flowability but also significantly enhanced its dispersibility, which is of great importance for its applications in food and pharmaceutical products [30]. Thus, reducing the particle size of spices not only optimizes the release of their volatile compounds but also enhances their bioavailability and functional properties in various applications.

### 3.2. Analysis of Volatile Compounds in Spices

**Figure 1:**
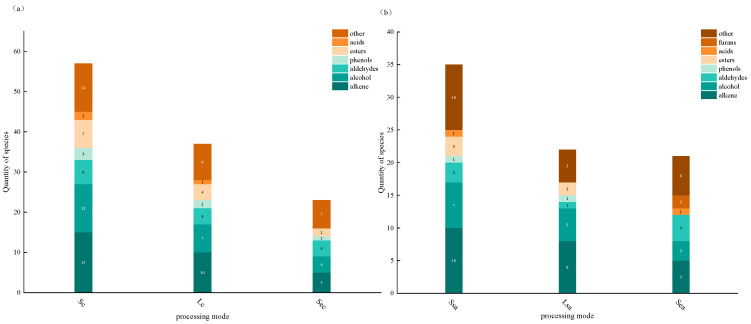
(**a**) shows the stacked distribution of flavor type counts in cinnamon under different treatments (Sc: general crushing; Lc: nano-vacuum collision; Sec: steam explosion). (**b**) shows the stacked distribution of flavor type counts in Illicium verum under different treatments (Ssa: general crushing; Lsa: nano-vacuum collision; Sea: steam explosion).

To better understand the volatile active compounds in cinnamon and star anise subjected to three different processing methods, GC–MS analysis was performed. The results are summarized in Table 2 and Table 3. In natural cinnamon, 53 volatile compounds were detected, whereas 39 compounds were identified after vacuum nano-collision treatment, and only 21 compounds were found following steam explosion. Similarly, in star anise, 35 volatile compounds were identified in the natural sample, 20 after nano-collision treatment, and 16 after steam explosion. Figure 1 shows the distribution of volatile compounds across treatments. Overall, terpenes were the predominant volatiles in both spices, followed by alcohols. Among the three processing methods, the ground (natural) spices contained the greatest number of volatiles, followed by vacuum nano-collision–treated samples, indicating that the total number of volatile compounds decreased after processing.

Interestingly, in star anise treated with vacuum nano-collision, the concentrations of major flavor compounds, such as limonene, eucalyptol, and linalool, increased significantly, and the key aroma compound anethole remained at a high concentration (512.74 μg/kg). While vacuum nano-collision was highly effective for star anise, traditional grinding remained slightly superior for preserving the key compound in cinnamon, suggesting that optimization of the nano-collision process may be required for this spice. In contrast, steam explosion not only decreased the overall number of volatiles but also promoted the formation of thermal degradation products, such as furfural, cyclohexene, and m-chlorobenzoic acid. For cinnamon, cinnamaldehyde, the main aroma compound, was abundant in all three treatments, with the highest content in natural samples (1526.43 μg/kg), followed by vacuum nano-collision (1493.45 μg/kg), and markedly reduced after steam explosion (526.78 μg/kg), indicating that high temperature and pressure caused significant volatilization or partial degradation. Additionally, 2′-methoxycinnamaldehyde was detected in all treatments with relatively stable concentrations. The differences observed between cinnamon and star anise under the three processing methods can be explained by the distinct chemical composition and thermal stability of their dominant volatile constituents. Cinnamon primarily contains aromatic aldehydes such as trans-cinnamaldehyde and 4′-methoxycinnamaldehyde, along with alcohols and terpenes (e.g., α-terpineol and eugenol). These aldehydes are highly volatile and thermally labile; therefore, steam explosion resulted in extensive degradation, and even under vacuum nano-collision conditions, localized pressure and frictional heat caused partial volatilization and oxidation, leading to slightly lower cinnamaldehyde content than in natural samples.

In contrast, star anise is rich in terpenes and phenylpropanoids such as anethole, limonene, eucalyptol, and linalool, which possess greater thermal and chemical stability. Vacuum nano-collision effectively disrupted oil glands and enhanced the dispersion and release of these aroma compounds under low-temperature conditions (5–10 °C), thus increasing their detectable concentrations without causing notable thermal degradation. However, steam explosion led to the decomposition of these volatiles and the generation of undesirable byproducts, confirming that excessive thermal stress promotes breakdown reactions.

Overall, the differential responses of cinnamon and star anise to processing treatments stem from the inherent thermal stability of their dominant flavor compounds: aromatic aldehydes in cinnamon are more heat-sensitive, whereas the terpenes and phenylpropanoids in star anise are relatively stable and can even be enhanced through controlled nano-collision processing.

### 3.3. Effects of Processing Methods on Key Volatile Compounds of Spices

The importance of key aroma-active compounds is typically evaluated by calculating their odor activity values (OAVs), rather than solely based on their individual concentrations [31]. It is also important to consider the odor thresholds of these aroma compounds. Generally, compounds with OAV ≥ 1 contribute significantly to the overall flavor, and higher OAVs indicate a greater contribution of the compound to the aroma profile.

**Figure 2:**
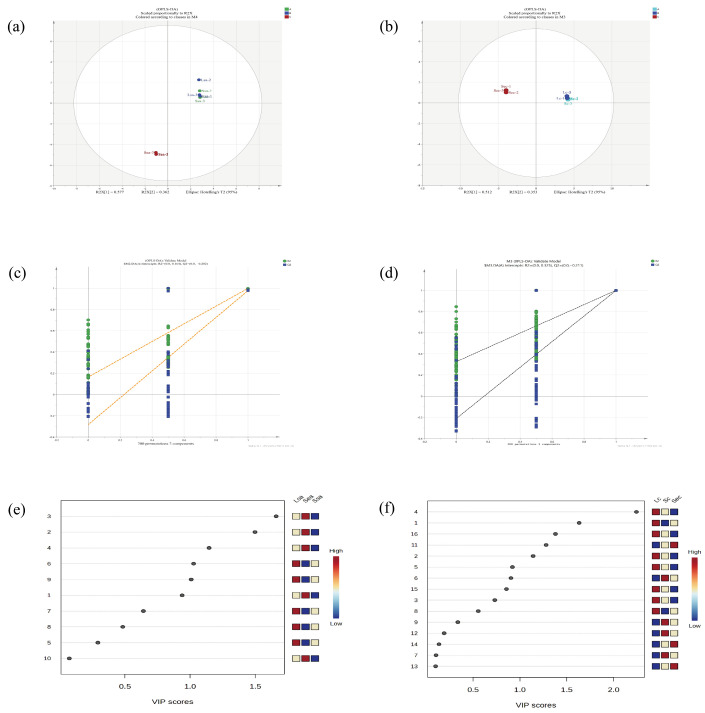
OPLS-DA analyses of key flavor compounds in star anise and cinnamon under three processing methods: (**a**,**b**) score plots, (**c**,**d**) model validation plots, and (**e**,**f**) VIP score plots. Lsa, Ssa, and Sea denote star anise samples processed by conventional grinding, vacuum nano-collision, and steam explosion, respectively; Lc, Sc, and Sec indicate the corresponding cinnamon samples.

Table A1 and Table A2 (refer to Appendix A and Appendix B) present the OAVs of volatile compounds in cinnamon and star anise, respectively, for the three different processing methods. Only compounds with OAV > 1 were considered as key aroma-active compounds. In cinnamon, both the ground (natural) and vacuum nano-collision–treated samples contained 13 key aroma compounds, with cinnamaldehyde exhibiting the highest OAV, dominating the characteristic cinnamon aroma, spiciness, and woody notes. After steam explosion treatment, only eight key compounds remained, with α-terpineol, camphor, β-longipinene, Δ-juniperene, T-linalool, and trans-oleic acid no longer detected. In star anise, slightly ground and vacuum nano-collision–treated samples contained ten key aroma compounds, with anethole showing the highest OAV and serving as the core contributor to the characteristic sweet and spicy aroma of star anise. Steam explosion treatment resulted in the loss of several compounds, including isoeugenol, α-terpineol, cinnamyl acetate, limonene, and methyl cinnamate. Figure 2 presents the OPLS-DA score plots, model validation plots, and VIP score plots of the key aroma compounds in star anise and cinnamon under different processing methods. Panels a and b correspond to the OPLS-DA score plots for star anise and cinnamon, respectively. OPLS-DA simplifies complex datasets to visualize high-dimensional patterns and assists in discriminating potential metabolites associated with changes in flavor profiles [32,33], OPLS-DA can also effectively discriminate differences between sample groups even when the variations among groups are subtle [34], in the figure, the combined values of R^2^X_1_ and R^2^X_2_ exceed 85%, indicating a high explanatory power of the model [35]. In the score plots, star anise and cinnamon treated by conventional grinding and vacuum nano-collision technology are located in the same quadrant, whereas samples subjected to steam explosion are distributed in a different quadrant. This distribution pattern indicates that the differences in key aroma-active compounds between the conventional grinding and nano-collision treatments are minimal, while steam explosion significantly alters the composition of key volatile compounds, resulting in marked differences from the other treatment groups. Panels e and f show the VIP score plots for the key aroma compounds in star anise and cinnamon, respectively. Compounds with VIP ≥ 1 are considered to have a significant contribution to the flavor, and higher VIP values indicate a greater contribution of the compound to distinguishing differences among samples in the model [21]. In star anise, the compounds with VIP values greater than 1 are numbered 3, 2, 4, 6, and 9, corresponding to anethole, 4-terpineol, isoeugenol, α-terpineol, and methyl cinnamate, respectively. In cinnamon, compounds with VIP > 1 are numbered 4, 1, 16, 11, and 2, representing 2′-methoxycinnamaldehyde, cinnamaldehyde, α-ylangene, Δ-juniperene, and 4-terpineol, respectively. Panels c and d show the OPLS-DA model validation plots for star anise and cinnamon. The model exhibited excellent fit, with R^2^X = 0.998 and R^2^Y = 1. After 200 permutation tests, Q^2^ intersected the *y*-axis below zero, indicating that the model is reliable and not overfitted. Therefore, the screening of the above-mentioned key aroma compounds is valid and feasible.

### 3.4. PCA-Based Analysis of Major Compound Differences in Spices

**Figure 3:**
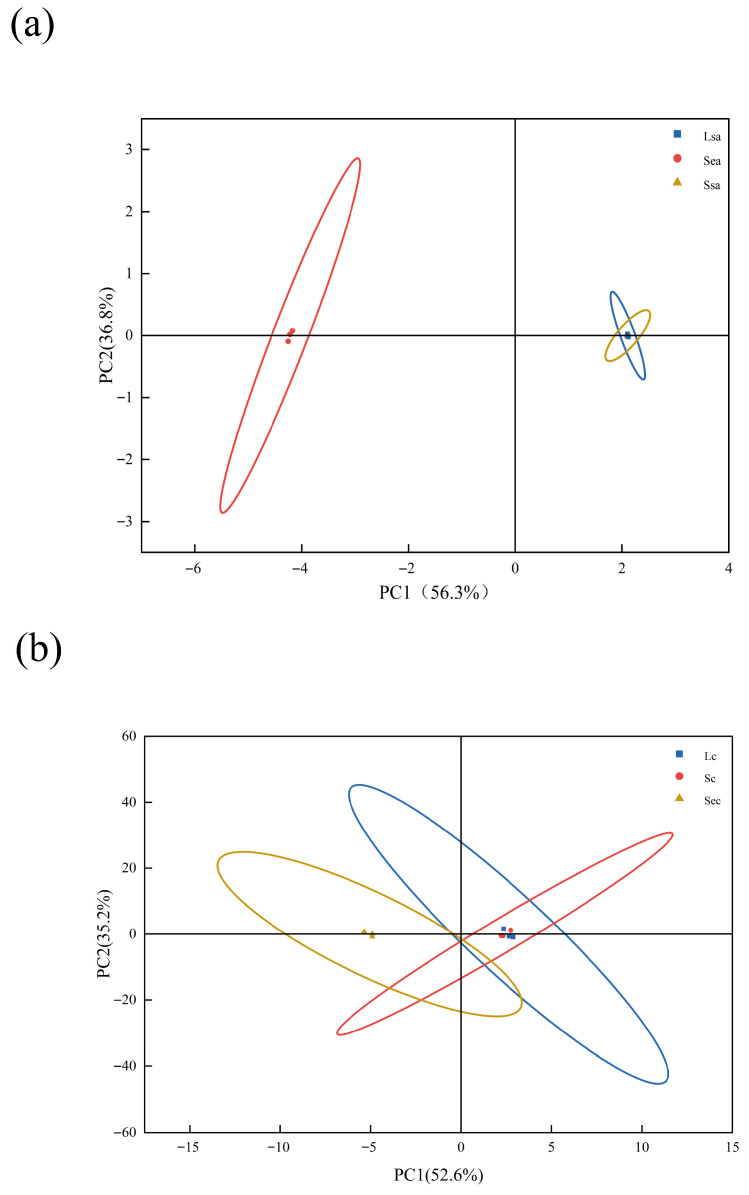
PCA plots of key flavor compounds in star anise and cinnamon under three processing methods: (**a**) Lsa, Ssa, and Sea denote star anise samples processed by conventional grinding, vacuum nano-collision, and steam explosion, respectively; (**b**) Lc, Sc, and Sec indicate the corresponding cinnamon samples.

The key flavor compounds of the spices were identified through calculation and screening, and further analysis of the principal component differences between the two spices under different treatments was conducted. Principal Component Analysis (PCA) was applied to reduce the dimensionality of the data. Figure 3 shows the PCA plot of key flavor compounds for the two spices under three treatment methods.

In Figure 3a, Lsa, Ssa, and Sea represent star anise treated by conventional crushing, vacuum nano-collision, and steam explosion, respectively. In Figure 3b, Lc, Sc, and Sec denote cinnamon subjected to the same three treatments: conventional crushing, vacuum nano-collision, and steam explosion. In both plots, the cumulative variance explained by PC1 and PC2 exceeded 80%, indicating that the extracted principal components effectively captured the major variability among the samples, demonstrating strong representativeness and explanatory power [21].

As observed from the distribution of sample points in the plot, the samples treated by conventional crushing and vacuum nano-collision technology are located within the same quadrant, with their points relatively concentrated and close to each other. This indicates that the overall aroma characteristics of the spices processed by these two methods are similar. In contrast, the sample points from the steam explosion treatment group are situated alone in a different quadrant, significantly distant from those of the other two treatment groups. This spatial separation suggests that steam explosion considerably altered the volatile flavor composition of the spices, resulting in a distinct flavor profile that differs markedly from those of the conventional crushing and nano-collision groups. These PCA findings are consistent with the results obtained from the OPLS-DA analysis.

### 3.5. Heatmap Clustering of Key Flavor Compounds Across Processing Methods

**Figure 4:**
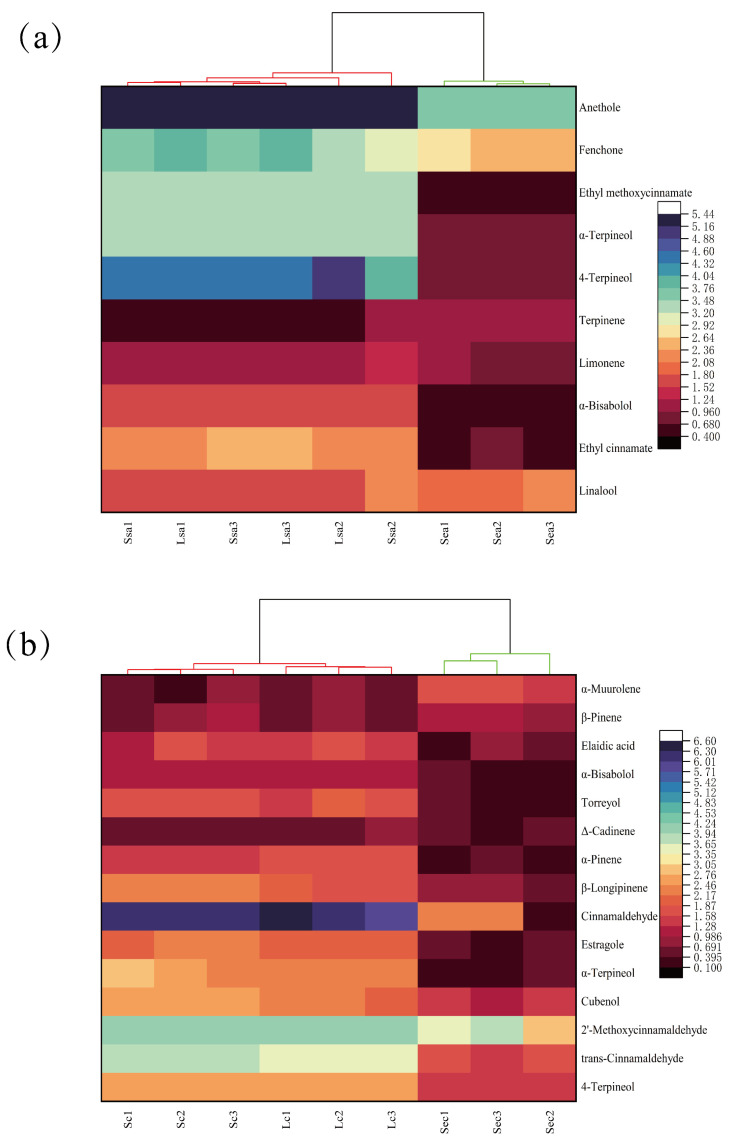
Hierarchical clustering heatmaps of key flavor compounds in star anise and cinnamon under three processing methods: (**a**) star anise samples (Lsa: conventional grinding; Ssa: vacuum nano-collision; Sea: steam explosion); (**b**) cinnamon samples (Lc: conventional grinding; Sc: vacuum nano-collision; Sec: steam explosion).

**Figure 5:**
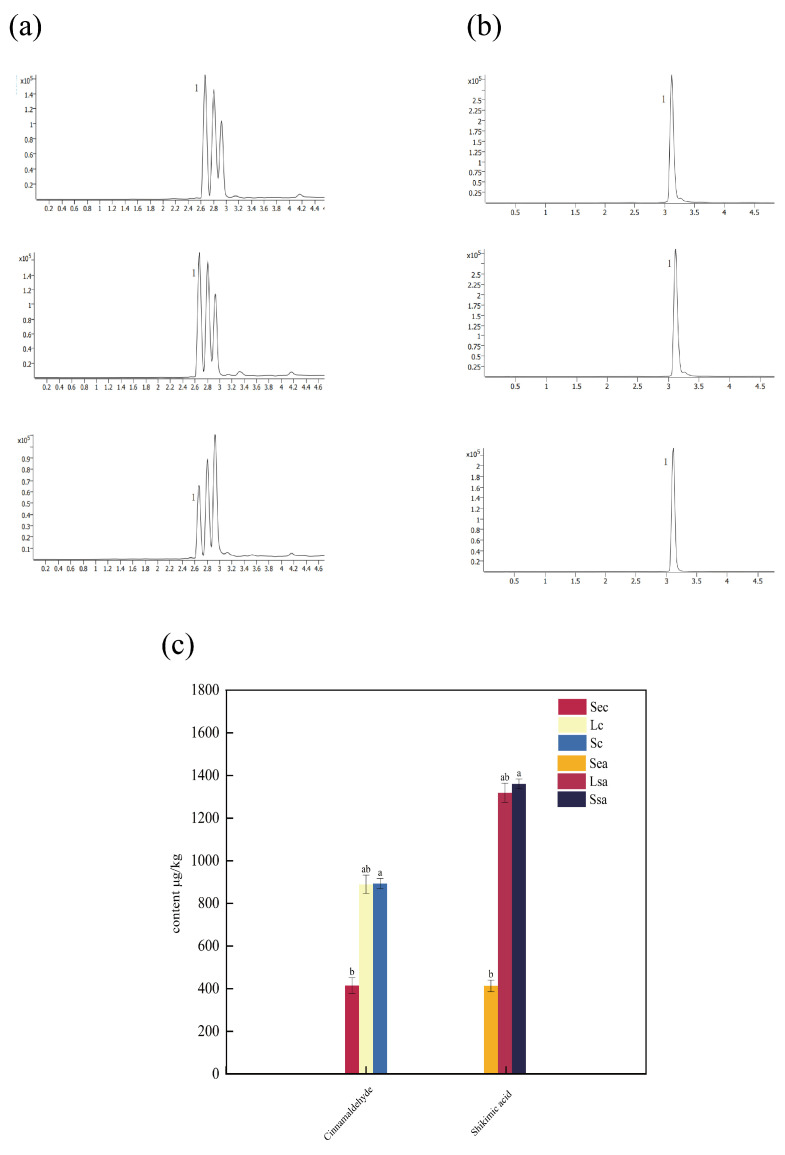
Chromatograms and variations in the contents of major active compounds in star anise and cinnamon under different processing methods: (**a**) chromatograms of shikimic acid in star anise (Lsa: conventional grinding; Ssa: vacuum nano-collision; Sea: steam explosion); (**b**) chromatograms of cinnamaldehyde in cinnamon (Lc: conventional grinding; Sc: vacuum nano-collision; Sec: steam explosion); (**c**) variations in the content of shikimic acid in star anise and cinnamaldehyde in cinnamon under the three treatments. Different letters (a,b) within the same row indicate statistically significant differences at *p* < 0.05.

Cluster hierarchical heatmap analysis was employed to identify differences in key flavor compounds among star anise and cinnamon under different treatment methods. As shown in Figure 4a, the heatmap illustrates the content composition of key flavor substances in star anise subjected to various treatments. Lsa, Ssa, and Sea represent star anise samples treated by conventional crushing, vacuum nano-collision, and steam explosion, respectively. Significant differences in the distribution of flavor components were observed among the samples. Based on the similarity of flavor profiles, the samples could be broadly divided into two major clusters: one primarily consisting of Lsa and Ssa-treated samples, and the other comprising Sea-treated samples. This clustering result indicates that the flavor profiles of star anise treated with conventional crushing (Lsa) and vacuum nano-collision (Ssa) were relatively similar, while steam explosion (Sea) led to significant alterations in flavor composition. Specifically, the relative abundance of key compounds such as anethole, fenchone, and ethyl methoxycinnamate was generally higher in the Lsa and Ssa groups but decreased after Sea treatment. Figure 5b presents the cluster heatmap of key flavor compounds in cinnamon under different treatments. As in Figure 5a, the samples were divided into two main clusters: one dominated by vacuum nano-collision (Lc) and conventional crushing (Sc) treatments, and the other consisting of steam explosion (Sec) treated samples. The key flavor compounds in the Lc and Sc groups exhibited clear differences compared to the Sec group. Moreover, the content of major flavor substances in cinnamon decreased noticeably after Sec treatment. The clustering analysis further confirmed this trend, as the steam-explosion-treated samples formed a distinct separate cluster, clearly diverging from the conventional crushing and nano-collision groups. This reinforces the conclusion that steam explosion treatment significantly affects the flavor characteristics of the spices.

### 3.6. Analysis of Major Bioactive Component Contents in Spices

Spices not only impart unique flavors to food but are also rich in various physiologically active compounds. Shikimic acid and cinnamaldehyde, representative non-volatile flavor substances in star anise and cinnamon, contribute to aroma formation but are also closely associated with functional properties such as antioxidant and antibacterial activities. In addition to analyzing volatile flavor compounds in the spices, this study used high-performance liquid chromatography-mass spectrometry (LC-MS) to quantitatively evaluate the major non-volatile constituents under different treatment methods. The results are shown in Figure 5. Figure 5a,b display the ion chromatograms of shikimic acid in star anise and cinnamaldehyde in cinnamon, respectively, under each treatment condition. Figure 5c illustrates the variation in the levels of these key compounds across different processing methods. As shown, the contents of shikimic acid and cinnamaldehyde in star anise and cinnamon decreased significantly after steam explosion treatment, measuring only 457.3 ng/mL and 414.7 ng/mL, respectively. In contrast, traditional crushing and nano-collision treatment resulted in notably higher levels of shikimic acid (1510.1 ng/mL and 1463.8 ng/mL, respectively) and cinnamaldehyde (893.1 ng/mL and 889.1 ng/mL, respectively). A substantial difference in content was observed between these two methods and steam explosion. Samples treated with traditional crushing and vacuum nano-collision showed no significant difference in the levels of major functional compounds, and both performed better than the steam explosion treatment. In conclusion, steam explosion treatment is harmful to preserving key constituents in spices, while vacuum nano-collision technology shows excellent performance in maintaining flavor-active components. These results emphasize its potential as a practical alternative to natural solid spices for use in marinated meat products.

### 3.7. E-Tongue Assessment of Spice-Braised Chicken Thighs Under Three Treatment Methods

**Figure 6:**
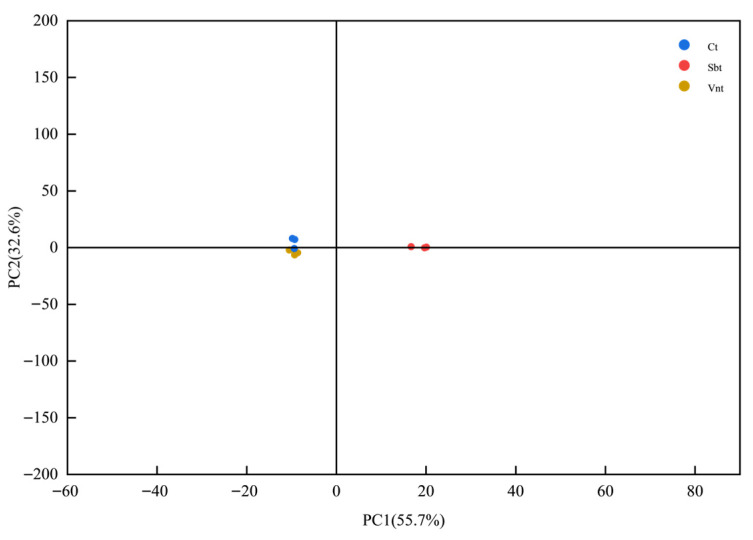
Principal Component Analysis (PCA) of taste compounds in braised chicken thighs marinated with spices processed by different methods. Treatments: CT, natural spices; Sbt, steam-exploded spices; Vnt, vacuum nano-collision spices.

Although the specific physical meaning of each principal component (PC) is not directly defined, in the context of electronic tongue analysis, PC1 and PC2 generally represent the main directions of variance in the overall taste response signals. PC1 usually corresponds to the most significant differences in overall taste intensity or balance among the samples, while PC2 reflects secondary variations, such as differences in individual taste attributes (e.g., sourness, saltiness, or umami). Therefore, clear separation or clustering of sample groups along these axes indicates that the electronic tongue can effectively distinguish the comprehensive taste characteristics resulting from different spice treatment methods. To evaluate the effects of three treatment methods on the flavor characteristics of the final product, marinated chicken drumsticks prepared with spices treated by traditional grinding (CT), vacuum nano-impact technology (VNT), and steam explosion treatment (SBT) were analyzed using an electronic tongue to assess their taste profiles and compare the overall taste differences among the three treatments. The electronic tongue, based on electrochemical sensing technology, enables objective and stable characterization of food flavor profiles, effectively reducing subjective bias associated with traditional sensory evaluation [29]. The results are shown in Figure 6. Principal Component Analysis (PCA) was applied to visualize the data, and the cumulative contribution rate of PC1 and PC2 exceeded 85%, indicating that the model effectively captured the overall differences among the sample groups. As shown in the principal component analysis (PCA) score plot, the differences in overall taste among the three groups were illustrated. The sample points of the CT and VNT groups were highly clustered, with short spatial distances and no clear separation trend, indicating that the overall taste differences between the marinated chicken drumsticks of these two groups were not significant. In contrast, the SBT group was distributed in different quadrants from the CT and VNT groups, with relatively large distances between sample points, indicating significant differences in overall taste characteristics. The results showed that spices treated with vacuum nano-collision technology imparted flavors to the chicken drumsticks that were largely similar to those of traditional spices, whereas the flavors conferred by spices subjected to steam explosion treatment were markedly different from the other two groups.

## 4. Conclusions

This study systematically compared the differences in physical properties and volatile and non-volatile flavor compound contents of star anise and cinnamon under different treatment methods and analyzed their taste characteristics using an electronic tongue. The results demonstrated that spices treated with vacuum nano-collision exhibited superior solubility compared to other groups, achieved a nanoscale particle size, and enabled faster flavor release. The treatment methods significantly influenced the types and contents of flavor substances, with steam explosion causing the most substantial loss of flavor compounds. In comparison, both conventional crushing and vacuum nano-collision treatments performed well in retaining key volatile components (such as anethole in star anise and cinnamaldehyde in cinnamon) and major non-volatile constituents (e.g., shikimic acid and cinnamaldehyde), with minimal differences in taste profiles, as indicated by electronic tongue analysis. Integrating flavor compound quantification and taste sensing results, the vacuum nano-collision technique demonstrated potential advantages in enhancing key flavor substance concentration, achieving uniform release, and maintaining spice quality. The findings suggest that spices treated by vacuum nano-collision can partially replace natural spices in marinated meat production. This study provides valuable insights for developing novel spice processing technologies and their application in meat products, while also proposing new strategies for low-carbon, low-energy industrial production of marinated foods.

## Figures and Tables

**Table 1:** The detection results of various indicators of different forms of spices.

Various Indicators	The Name of the Spice					
	Lsa	Ssa	Sea	Lc	Sc	Sec
MC%	78 ± 2.37 ^a^		68.63 ± 3.11 ^b^	75 ± 3.51 ^a^		66.57 ± 2.66 ^b^
WSI/%	64.96 ± 1.12 ^c^	93.41 ± 2.83 ^a^	78.36 ± 1.28 ^b^	93.5 ± 0.71 ^a^	71.94 ± 2.09 ^bc^	75.37 ± 2.29 ^b^
GS/nm	13.65 ± 0.22 ^c^		879.33 ± 3.37 ^b^	10.40 ± 0.65 ^c^		935.19 ± 2.56 ^a^

Note: MC in the table is water content, WSI is solubility and GS is particle size. Lsa, Ssa, and Sea represent star anise samples treated by conventional grinding, vacuum nano-collision technology, and steam explosion technology, respectively. Lc, Sc, and Sec represent cinnamon samples subjected to the same respective treatments, values are presented as mean ± standard deviation. Different letters (a, b, etc.) within the same row indicate statistically significant differences at *p* < 0.05.

**Table 2:** Volatile compounds and their concentrations in cinnamon bark.

Flavor Ingredient Name	Concentration (μg/kg)		
	Sc	Lc	Sec
Limonene	40.02 ± 0.0009 ^ab^	50.665 ± 0.0009 ^ab^	3.4465 ± 0.0014 ^a^
Eucalyptus oil alcohol	1.6537 ± 0.0008 ^b^	1.6537 ± 0.008 ^b^	8.5000 ± 0.0016 ^a^
linalool	1.5921 ± 0.0006 ^a^	/	/
L-camphor	3.8227 ± 0.0015 ^a^	/	/
2-Ziol	4.2784 ± 0.0013 ^a^	2.6359 ± 0.0009 ^b^	4.8319 ± 0.0013 ^a^
Cinnamyl	9.6144 ± 0.0008 ^b^	8.7559 ± 0.0012 ^b^	38.4309 ± 0.042 ^a^
Synthetic dextrotron	2.6359 ± 0.0006 ^a^	/	/
4-terpene alcohols	3.1968 ± 0.0015 ^a^	2.768 ± 0.0003 ^ab^	1.4524 ± 0.0002 ^b^
α-terpineol	76.5634 ± 0.0046 ^a^	65.8552 ± 0.0037 ^b^	/
Myristicin	1.5613 ± 0.0003 ^a^	1.5950 ± 0.0002 ^a^	/
Trans-cinnamaldehyde	72.9396 ± 0.0064 ^a^	68.8068 ± 0.0056 ^ab^	33.8468 ± 0.0021 ^b^
3-Phenylpropanol	11.4955 ± 0.0012 ^a^	/	10.9805 ± 0.0008 ^a^
3-Methoxybenzaldehyde	0.8736 ± 0.0001 ^a^	/	/
Cinnamic alcohol	1526.4309 ± 0.1037 ^a^	1493.4441 ± 0.1139 ^ab^	526.6724 ± 0.0763 ^b^
β-Caryophyllene	9.5144 ± 0.0018 ^a^	7.7356 ± 0.0013 ^b^	/
α-Phellandrene	6.4577 ± 0.0013 ^a^	6.9070 ± 0.0011 ^ab^	4.2469 ± 0.0008 ^b^
Eugenol	9.6144 ± 0.0011 ^b^	8.7560 ± 0.0015 ^b^	38.431 ± 0.0024 ^a^
(+)-Cyclic Alfalpene	8.0260 ± 0.0017 ^b^	/	14.1002 ± 0.0011 ^a^
γ-mulene	9.2908 ± 0.0019 ^a^	/	/
Longifoliene	5.5152 ± 0.0013 ^b^	8.3227 ± 0.0019 ^a^	7.8302 ± 0.0016 ^ab^
β-Caryophyllene	6.3813 ± 0.0015 ^b^	8.3882 ± 0.0017 ^a^	/
(±)-β-cobaene	5.0058 ± 0.0012 ^a^	/	/
Γ-juniperene	7.5718 ± 0.0016 ^a^	/	/
α-Rhythmene	7.5276 ± 0.0018 ^a^	1.9092 ± 0.0006 ^b^	/
Ethyl cinnamate	13.1002 ± 0.0023 ^a^	/	/
Guiaoxin is a form of xylol	14.3322 ± 0.0027 ^a^	/	/
Alpha-cobaene	7.2007 ± 0.0015 ^a^	/	/
α-Eylene	10.4180 ± 0.0011 ^a^	4.8114 ± 0,0013 ^b^	/
Valencian oranges	0.8736 ± 0.0001 ^a^	/	/
α-Caryophyllene	16.3934 ± 0.0029 ^a^	/	/
Delta-juniperene	88.1975 ± 0.0067 ^ab^	91.0855 ± 0.0087 ^a^	/
Dehydroleucene	8.2811 ± 0.0017 ^b^	42.6684 ± 0.0038 ^a^	/
4′-Methoxycinnamaldehyde	3.1968 ± 0.0006 ^a^	2.7685 ± 0.0002 ^ab^	1.4524 ± 0.0001 ^b^
Glycene	6.9697 ± 0.0013 ^a^	/	/
Melaleuca alcohol	7.6575 ± 0.0015 ^a^	/	/
Eggplant enol	77.5115 ± 0.0065 ^a^	66.2722 ± 0.0034 ^b^	43.9026 ± 0.0054 ^ab^
T-juniper alcohol	19.2349 ± 0.0026 ^a^	16.1339 ± 0.0122 ^a^	/
trans-β-Ocimene	48.4644 ± 0.0033 ^a^	41.4642 ± 0.0032 ^ab^	/
α-Bisabolol	30.4392 ± 0.0028 ^b^	35.0544 ± 0.0021 ^a^	/
Citron mellow	27.1747 ± 0.0069 ^a^	28.9666 ± 0.0031 ^b^	/
Bisabolol	9.6760 ± 0.0012 ^a^	9.8129 ± 0.0008 ^a^	/
eucalyptole enol	8.1477 ± 0.0014 ^a^	/	/
Ethyl p-methoxycinnamate	2.7395 ± 0.0012 ^a^	3.6324 ± 0.0013 ^a^	/
Dehydrolignolide	3.4878 ± 0.0015 ^a^	2.7873 ± 0.0009 ^a^	/
trans-Isoeugenol	0.4969 ± 0.0001 ^a^	0.5800 ± 0.0002 ^a^	/
Methyl trans-9-octadecaenoate	0.4358 ± 0.0001 ^a^	/	/
Phenethyl acetate	2.7675 ± 0.0013 ^a^	/	/
Perillalactone	2.8908 ± 0.0012 ^a^	/	/
Oleptyl	1.8210 ± 0.0009 ^a^	/	/
Trumpet tea alcohol	5.1833 ± 0.0016 ^b^	8.6547 ± 0.0019 ^a^	/
Green flowers and melaleuca alcohol	4.4191 ± 0.0013 ^a^	/	/
(-)-isolongol	4.1899 ± 0.0013 ^a^	/	/
geraniol	1.7216 ± 0.0008 ^a^	/	/
1-Indenone	/	1.4085 ± 0.0007 ^a^	/
n-propyl cinnamate	/	1.4495 ± 0.0005 ^a^	/
(+)-Alfalpene	/	2.9660 ± 0.0009 ^a^	/
α-cypressene	/	2.8388 ± 0.0011 ^a^	/
coumarin	/	41.5196 ± 0.0039 ^b^	68.2397 ± 0.0056 ^a^
Bicyclic geraniene	77.5116 ± 0.0023 ^a^	66.2721 ± 0.0019 ^ab^	43.9026 ± 0.0016 ^b^
Alpha-di-dehydrocalamus alem	/	16.3934 ± 0.0024 ^a^	/
mannitol	/	10.2691 ± 0.00019 ^a^	/
cedar alcohol	/	9.6841 ± 0.0018 ^a^	/
Alpha-calamus alcohol	/	2.7395 ± 0.0011 ^a^	/
α pinene	76.5633 ± 0.0025 ^a^	65.8551 ± 0.0021 ^ab^	/
Isopropyltoluene	/	/	1.2516 ± 0.0007 ^a^
anethole	/	/	13.5331 ± 0.0008 ^a^
Aninene	/	/	1.3167 ± 0.0007 ^a^
α-Curcumene	/	/	148.9596 ± 0.0159 ^a^
Methyl linoleate	/	/	17.0585 ± 0.0021 ^a^
2′-Methoxycinnamaldehyde	291.4772 ± 0.0123 ^a^	284.0859 ± 0.0118 ^ab^	251.8005 ± 0.0112 ^b^

Note: / indicates that the compound is not detected, values are presented as mean ± standard deviation. Different letters (a, b, etc.) within the same row indicate statistically significant differences at *p* < 0.05.

**Table 3:** Volatile compounds and concentrations in star anise.

Flavor Ingredient Name	Concentration (μg/kg)		
	Ssa	Lsa	Sea
Cressellin	1.8329 ± 0.0006 ^b^	2.1545 ± 0.0009 ^b^	12.0154 ± 0.0024 ^a^
pinene	19.6048 ± 0.0012 ^ab^	23.2812 ± 0.0031 ^a^	14.4500 ± 0.0026 ^b^
Cypress	14.2616 ± 0.0023 ^a^	/	/
α-thujagone	12.1143 ± 0.0021 ^a^	/	/
4-Isopropyltoluene	9.6621 ± 0.0015 ^b^	28.1913 ± 0.0038 ^a^	/
Citral	45.4050 ± 0.0056 ^b^	58.6355 ± 0.0126 ^a^	/
Eucalyptus oil alcohol	38.8992 ± 0.0045 ^b^	100.1467 ± 0.0089 ^a^	/
γ-terpinene	4.7048 ± 0.0011 ^c^	68.7413 ± 0.0076 ^a^	11.0732 ± 0.0021 ^b^
linalool	28.9180 ± 0.0014 ^ab^	32.6823 ± 0.0041 ^a^	18.0024 ± 0.0028 ^b^
2-Ziol	3.6147 ± 0.0012 ^a^	/	/
4-terpineol	35.5111 ± 0.0016 ^a^	38.0329 ± 0.0044 ^a^	33.1679 ± 0.0042 ^b^
α-terpineol	33.9314 ± 0.0043 ^ab^	38.6565 ± 0.0048 ^a^	/
4-Allyl anisole	7.9823 ± 0.0089 ^a^	7.2044 ± 0.0226 ^a^	8.3013 ± 0.0654 ^a^
3-Methoxybenzaldehyde	117.1289 ± 0.0108 ^a^	/	/
Methyl chavicol	498.8312 ± 0.031 ^ab^	512.738 ± 0.036 ^a^	331.272 ± 0.0025 ^b^
α-Lymphatic solene	58.3498 ± 0.0067 ^b^	77.8220 ± 0.0061 ^a^	/
β-Caryophyllene	49.8234 ± 0.0056 ^b^	108.4960 ± 0.0082 ^a^	56.4314 ± 0.0062 ^b^
α-cis-bo-limonene	23.0367 ± 0.0038 ^c^	81.0613 ± 0.0067 ^a^	48.8155 ± 0.0051 ^b^
α-Rhythmene	15.9122 ± 0.0026 ^a^	/	/
Cinnamyl acetate	79.4740 ± 0.0069 ^a^	/	/
α-Curcumene	33.5702 ± 0.0045 ^a^	/	/
β-Serene	81.7368 ± 0.0078 ^a^	/	/
γ juniperene	71.3309 ± 0.0065 ^a^	/	/
Delta-juniperene	89.7035 ± 0.0071 ^a^	27.6374 ± 0.0031 ^b^	/
Elemiol	13.4937 ± 0.0023 ^a^	/	/
Neroli tertiary alcohol	63.2022 ± 0.0059 ^a^	17.1422 ± 0.0029 ^b^	/
Dianthus plain	51.5094 ± 0.0042 ^a^	/	/
T-ylang ylang alcohol	48.4736 ± 0.0057 ^a^	/	/
Cyclodiene	59.9278 ± 0.0048 ^a^	/	/
α-Bisabolol	47.325 ± 0.0071 ^a^	51.783 ± 0.0038 ^a^	/
Methyl cinnamate	208.7260 ± 0.0172 ^a^	195.6375 ± 0.0098 ^ab^	/
palmitic acid	54.2902 ± 0.0062 ^a^	/	/
3-Cruthene	/	/	13.4096 ± 0.0026 ^a^
terpinlene	5.6103 ± 0.0006 ^b^	6.3587 ± 0.0009 ^b^	12.0069 ± 0.0029 ^a^
furfural	/	/	21.35831 ± 0.0035 ^a^
5-Methylfurfural	/	/	26.40385 ± 0.0031 ^a^
cyclohexene	/	/	73.23692 ± 0.0082 ^a^
m-chlorobenzoic acid	/	/	11.17162 ± 0.0008 ^a^
3-Methoxybenzaldehyde	/	/	139.4462 ± 0.0088 ^a^
Myrtenol	7.5246 ± 0.0037 ^a^	7.1420 ± 0.0025 ^a^	/

Note: / indicates that the compound is not detected, values are presented as mean ± standard deviation. Different letters (a, b, etc.) within the same row indicate statistically significant differences at *p* < 0.05.

## Data Availability

The original contributions presented in this study are included in the article. Further inquiries can be directed to the corresponding authors.

## Appendix A

**Table A1:** OAVs of key flavor compounds in cinnamon bark.

Flavor Compound Name	Flavor Description	Threshold	OAV		
			Sc	Lc	Sec
Eugenol	Spicy, sweet, woody aroma	2	4.8072 ± 0.0006 ^b^	4.3780 ± 0.0007 ^b^	19.2155 ± 0.0011 ^a^
4-Methoxycinnamaldehyde	Woody, citrus aroma	0.11	29.0618 ± 0.0017 ^a^	25.1679 ± 0.0014 ^ab^	13.2037 ± 0.0011 ^b^
trans-Cinnamaldehyde	Spicy, medicinal, woody bitterness	2	36.4698 ± 0.0025 ^a^	34.4034 ± 0.0023 ^a^	16.9234 ± 0.0008 ^b^
2′-Methoxycinnamaldehyde	Sweet-spicy, meaty, woody	7	41.6396 ± 0.0029 ^a^	40.5837 ± 0.0028 ^a^	35.9715 ± 0.0024 ^b^
Bicyclogermacrene	Woody, spicy, bitter-medicinal	3	25.8372 ± 0.0015 ^a^	22.0907 ± 0.0014 ^ab^	14.6342 ± 0.0009 ^b^
α-Pinene	Woody, spicy, bitter-medicinal	2.75	27.8412 ± 0.0017 ^a^	23.9473 ± 0.018 ^ab^	/
Myristicin	Piney, camphor, minty	0.075	20.8179 ± 0.0018 ^a^	21.2672 ± 0.0019 ^a^	/
Cinnamic alcohol	Piney, camphor, minty	2.39	638.674 ± 0.0035 ^a^	624.872 ± 0.0032 ^a^	220.365 ± 0.0012 ^b^
β-Caryophyllene	Minty aroma	0.4	23.7860 ± 0.0018 ^a^	19.3389 ± 0.0009 ^ab^	/
α-Phellandrene	Cinnamic, spicy, woody	0.41	15.7506 ± 0.0011 ^a^	16.8464 ± 0.0012 ^a^	10.3582 ± 0.0008 ^b^
α-Cubebene	Bitter aroma	3.6	24.4993 ± 0.0015 ^a^	25.3015 ± 0.0019 ^a^	/
trans-β-Ocimene	Woody, spicy, floral	3	16.1548 ± 0.0015 ^a^	13.8214 ± 0.0014 ^b^	/
α-Bisabolol	Woody, spicy, medicinal	3	10.1464 ± 0.0009 ^a^	11.6848 ± 0.0010 ^a^	/
trans-Isoeugenol	Spicy, sweet, acidic-floral	0.039	12.7422 ± 0.0011 ^ab^	14.8718 ± 0.0012 ^a^	/
α-Curcumene	Fruity, sweet, floral aroma	9.01	/	/	16.5327 ± 0.0014 ^a^

Note: / indicates that the compound is not detected, values are presented as mean ± standard deviation. Different letters (a, b, etc.) within the same row indicate statistically significant differences at *p* < 0.05.

## Appendix B

**Table A2:** OAVs of key flavor compounds in star anise.

Flavor Compound Name	Flavor Description	Threshold	OAV		
			Ssa	Lsa	Sea
Linalool	Floral, woody aroma	2.38	12.1504 ± 0.0016 ^a^	13.7321 ± 0.0015 ^a^	7.5640 ± 0.0008 ^b^
Methyl chavicol	Sweet, herbal, bitter	1	498.8312 ± 0.0031 ^ab^	512.738 ± 0.0036 ^a^	331.272 ± 0.0025 ^b^
Myrtenol	Piney, spicy	0.2	37.6230 ± 0.0027 ^a^	35.7100 ± 0.0026 ^ab^	/
α-Terpineol	Woody pine aroma	2.75	12.3387 ± 0.0013 ^ab^	14.0569 ± 0.0014 ^a^	/
Cinnamyl acetate	Cinnamic, floral, spicy aroma	3.6	22.0761 ± 0.0027 ^a^	/	/
4-Terpineol	Minty, woody, herbal	1.1	32.2828 ± 0.0023 ^ab^	34.5754 ± 0.0026 ^a^	30.1526 ± 0.0019 ^ab^
α-Bisabolol	Woody, spicy, herbal-medicinal aroma	3	15.7750 ± 0.0016 ^ab^	17.2610 ± 0.0013 ^a^	/
Terpinolene	Piney, woody aroma	1	5.6103 ± 0.004 ^b^	6.3587 ± 0.007 ^b^	12.0069 ± 0.0012 ^a^
Citral	Citrus, herbal aroma	5	9.0810 ± 0.0008 ^ab^	11.7271 ± 0.0012 ^a^	/
Methyl cinnamate	Spicy, sweet, herbal	5	41.7452 ± 0.0032 ^a^	39.1275 ± 0.0023 ^a^	/

Note: / indicates that the compound is not detected, values are presented as mean ± standard deviation. Different letters (a, b, etc.) within the same row indicate statistically significant differences at *p* < 0.05.
